# Acute Gastritis in Children

**Published:** 1889-12

**Authors:** T. M. Wilson

**Affiliations:** Thornton, Texas


### CORRESPONDENCE.

### ACUTE GASTRITIS IN CHILDREN.

THORNTON, TEXAS, NOV. 24, 1889.

*Ed. Daniel's Texas Medical Journal:*

I have moved from Kosse to Thornton, Texas; moved 31st ult. My baby boy, seven months and seven days old, died yesterday morning of acute gastritis, after an illness of thirteen days. We tried almost all the known remedies, or remedies supposed to be good in such diseases, without checking the trouble. If you have anything special to give in such cases, please let me know. I lost my first child, at four months and eleven days old, with gastro-intestinal catarrh. Lost our third, a female, aged two months and twenty-seven days, with acute gastritis. So you can imagine I am getting very anxious about the matter. I had several other physicians with each child, but all to no success. Our second had two attacks, each of which followed an attack of dengue. The first attack was at the age of nine or ten months; the second a year later. Both attacks seemed to be a sequel of dengue which probably accounts for the recovery. The last two that died had no perceptible premonitions, but the attack was ushered in with great pain, followed by a rising temperature, which, within a few hours, reached 102° to 104° F., and ranged between these extremes, except when brought down by antipyrine to 101° about twice during the attack of each child. The first child's attack came on more gradually, the bowels being first affected. The pain in every case seemed to never abate, notwithstanding camphorated tr. opii was given freely. The second child that died showed considerable brain trouble, which began to manifest itself during the third or fourth day of the attack.

The last one began to show cerebral complications about the sixth or seventh day of the attack, which symptoms gradually increased till death on the thirteenth day. Now, as I kept no notes of either case, I will add no more than to say, the symptoms in each case were such as given in our text books, especially "Smith on Diseases of Children." So was his treatment in the main followed, except that calomel was given in each case, especially at the beginning of the attack; quinine was given by inunction and enemata, but to no perceptible effect.

If convenient, let us have some suggestions from you, or anyone else who wishes to make any, or offer anything which he thinks is specially indicated. I have not written this for publication, but if you wish, you may give it to our profession.

Yours respectfully,

T. M. WILSON.

[We regret exceedingly to be unable to throw any light on the subject.—Gastro-intestinal catarrh, or the summer disease of children—that condition which usually attends teething, is one of the opprobria of medicine; there is no specific. No one course of treatment will promise a cure in the large majority of cases. The cases reported here were not of that character, however; all occurring in children under eight months old,—but, pathologically, we presume, they were the same. We invite a discussion of the subject,—throwing open our pages to those, who, unlike Dr. Wilson, have had success in the management of the trouble; and we beg to suggest, no more practically important subject could well be presented for discussion. Let us hear from you, gentlemen.—ED.]

